# Biologic Therapies in Sarcoidosis and Uveitis: A Review

**DOI:** 10.7759/cureus.9057

**Published:** 2020-07-07

**Authors:** Olisaemeka D Ogbue, Parul Malhotra, Radhika Akku, Thulasi Priya Jayaprakash, Safeera Khan

**Affiliations:** 1 Internal Medicine, California Institute of Behavioral Neurosciences & Psychology, Fairfield, USA; 2 Medicine, California Institute of Behavioral Neurosciences & Psychology, Fairfield, USA; 3 Internal Medicine, Punjab Institute of Medical Sciences, Ludhiana, IND

**Keywords:** infliximab, adalimumab, etanercept, sarcoidosis, chronic uveitis

## Abstract

Sarcoidosis and uveitis are chronic inflammatory conditions with potentially debilitating effects on quality of life. Steroids form the mainstay standard therapy in both conditions. Biologic agents are considered to be appropriate alternatives for treatment in steroid-refractory sarcoidosis and uveitis due to the role of tumor necrosis factor (TNF) in mediating the inflammatory cascade seen in both conditions. We performed a thorough literature search using PubMed to compare the extent of use, efficacy, and safety profile of individual anti-TNF agents in the management of these conditions. Our review consists of two systematic reviews with meta-analysis, thirteen observational studies, and fifteen case series/reports. Infliximab had the widest range of organ-system usage in extra-pulmonary sarcoidosis but is equivalent to adalimumab in terms of efficacy. In uveitis, adalimumab was found to be the most efficacious agent for maintaining disease remission in adults and children with chronic non-infectious uveitis. Etanercept was neither used widely, nor was it efficacious in the management of either condition. In terms of safety profile, biologic agents were found to be well tolerated and have a similar safety profile. More randomized clinical trials are needed to inform evidence-based use of biologic agents in these conditions.

## Introduction and background

Sarcoidosis is a systemic inflammatory disorder wherein the central feature is the formation and propagation of granulomas in affected organs. It has a prevalence of 10 per 100,000 people in the United States, with the incidence and prevalence being higher in African American populations compared to Asians, Hispanics, and Caucasians [1]. Pulmonary involvement in sarcoidosis is a cardinal feature, however, extra-pulmonary manifestations have been reported in 10-30% of cases [2]. Organ-systems, where sarcoid granulomas have been described, include bone in 3-5% of cases and 5-10% in neural tissue (neurosarcoidosis) [3,4]. Biopsy-confirmed sarcoid granulomas have also been reported in the gastrointestinal tract, lymphatic system, joints, heart, muscle, and skin, but epidemiological data are generally lacking.

 Uveitis refers to intraocular inflammation, which, in a strict sense, may involve the iris, ciliary body, or choroid [5]. This inflammation may be anterior, with the iris and ciliary body involved, posterior with the choroid, or both (panuveitis). Non-infectious uveitis (NIU) can be associated with systemic inflammatory disorders such as juvenile idiopathic arthritis, Behcet's syndrome, rheumatoid arthritis, including sarcoidosis (ocular sarcoidosis) and accounts for 10-20% of cases of blindness in developed countries [6]. The term idiopathic may be used to describe uveitis when there is no identifiable underlying etiology.

Tumour Necrosis Factor (TNF) is a participatory cytokine in many inflammatory disorders and plays a role in the formation & propagation of sarcoid granulomas [7]. The role of TNF as a pro-inflammatory cytokine, secreted primarily by macrophages, includes the triggering cell necrosis and apoptosis. TNF also induces the proliferation and differentiation of effector cells, which in turn mediate a wide variety of downstream inflammatory responses (secondary effect). Infliximab, adalimumab, and etanercept are amongst some of the commercially available agents directed against TNF with others being golimumab and certolizumab pegol. These agents can be referred to as biologics as they are derived from living organisms and their products or anti-TNF agents as they are primarily targeted to antagonize TNF and its effects. This article will focus on three agents: infliximab, adalimumab, and etanercept. The use of these agents has been associated with the occurrence of antibodies directed against them. However, data regarding the effect of these antibodies on drug efficacy or side effect profiles of these agents is lacking [8].

Corticosteroids are considered to be the mainstay and standard treatment in both sarcoidosis and chronic uveitis [9], however long-term steroid therapy of these chronic conditions is often associated with intolerable systemic side-effects, steroid resistance or both [10]. Experience in treatment with alternatives to steroids is limited due to the rarity of the conditions. In situations wherein sarcoidosis and chronic uveitis become refractory to steroids, biologics or anti-TNF agents are considered appropriate alternatives [9]. In steroid-resistant sarcoidosis, biologic agents are used in both pulmonary and extra-pulmonary locations, but results with individual agents have been mixed [9]. Experience with biologic agents lacks more so in extra-pulmonary sarcoidosis due to disease rarity in the population. In chronic uveitis, given the diverse possible etiologies, there is, at present, no widely accepted non-corticosteroid treatment protocol when uveitis becomes refractory to steroids. Anti-TNF usage is mostly off-label, and evidence supporting the use of specific agents is lacking [8]. The safety profile of anti-TNF agents in the management of extra-pulmonary sarcoidosis and chronic uveitis as monotherapy or combination therapy is also not well understood.

The purpose for this article is to review the literature to highlight the role of the common anti-TNF agents, infliximab, adalimumab, and etanercept, in the management of extra-pulmonary sarcoidosis and non-infectious uveitis, summarize key articles comparing the efficacy and safety profile of these agents as well as discuss possible areas for future research.

## Review

Methods

A thorough literature search was conducted using PubMed with infliximab, adalimumab, etanercept as keywords. These were used in combination with sarcoidosis and chronic uveitis. Search filters applied include the availability of full text and publishing within the last ten years. This initial search yielded 248 articles, but only 50 papers were shortlisted for abstract review after careful screening of articles based on relevance to the research question. Duplicate studies in review articles were excluded. Inclusion and exclusion criteria were applied during in-text screening, and 30 papers were chosen for the final review. Inclusion criteria helped include articles relevant to the research question with full text available published in English. Articles focused on pulmonary sarcoidosis were screened out using the exclusion criteria.

**Figure 1:**
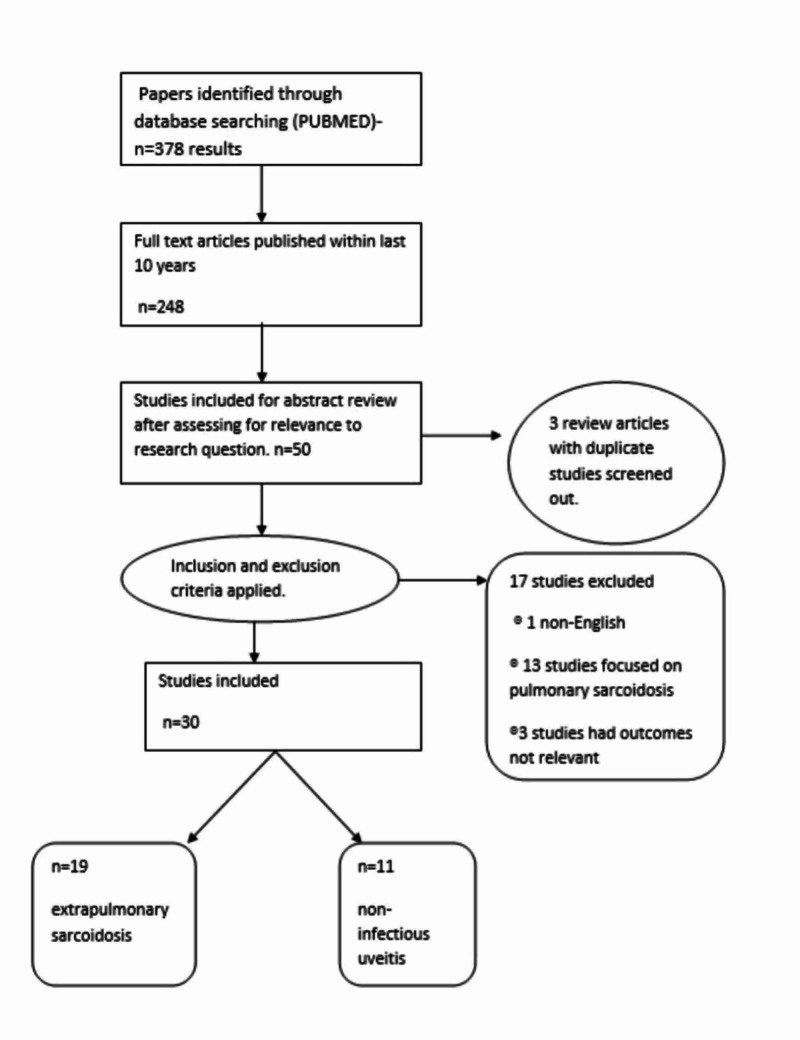
PRISMA Diagram Showing Selection of Data

Additional supportive references were included in the introduction and discussion sections. PRISMA diagram describing the selection of data is shown in Figure 1.

Results

Thirty articles included for the final review are composed of two systematic reviews with meta-analysis [11,12], fifteen case reports/series [13-27], and thirteen observational studies [1,3,4,9,28-37]. The two systematic reviews provided data on 692 patients. Leal et al. assessed the efficacy of anti-TNF agents in adult NIU while Simoncini et al. compared the efficacy of the three agents (infliximab, adalimumab, and etanercept) in pediatric NIU [11,12].

Discussion

Mechanism of Action of Anti-TNF Agents

**Figure 2:**
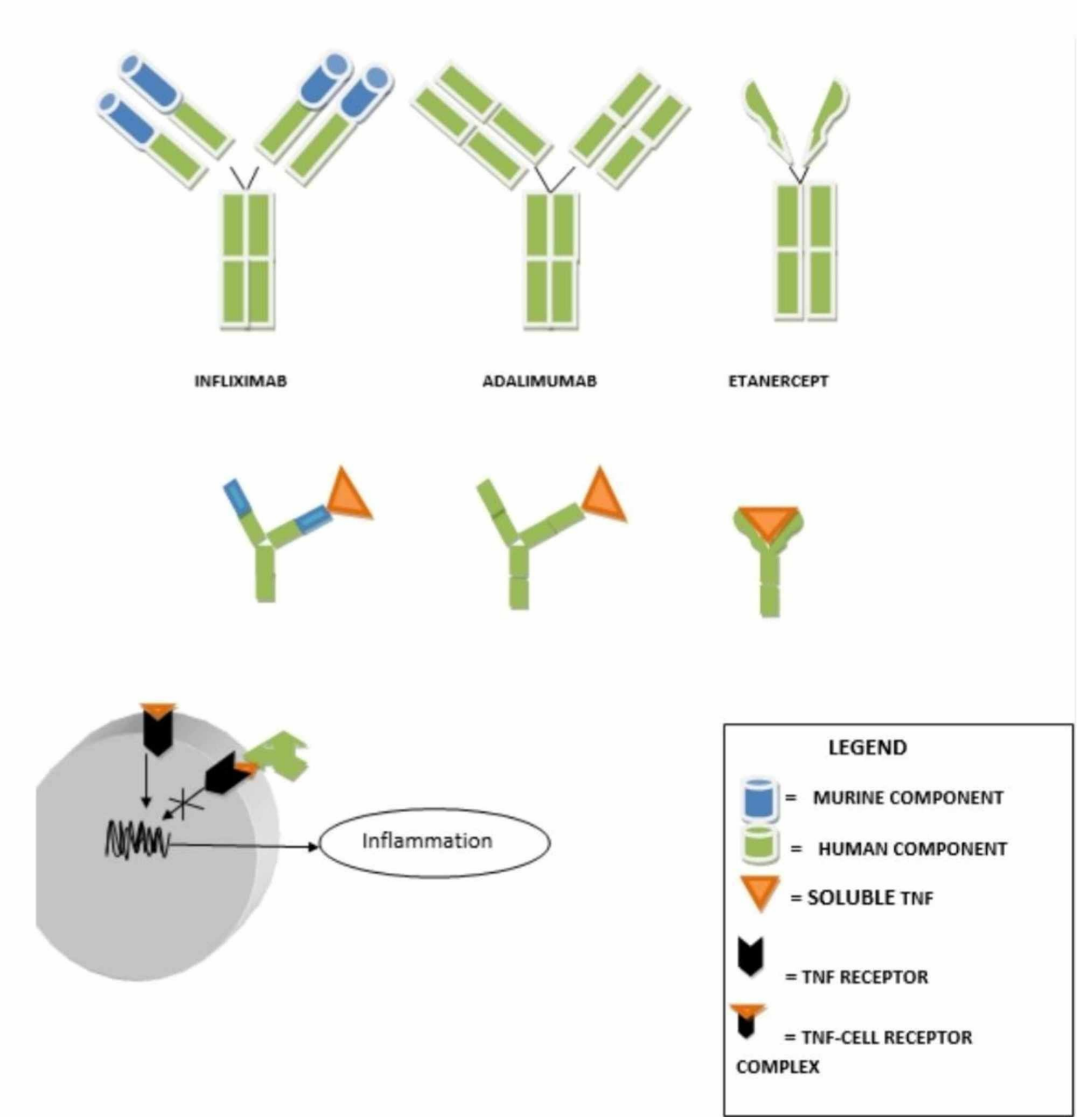
Mechanism of Action of Anti-TNF Agents TNF- Tumour Necrosis Factor

Adalimumab and infliximab have similar mechanisms of action by binding to soluble TNF-α, thereby rendering it biologically inactive. Adalimumab also causes lysis of inflammatory cells bound to TNF by interacting with the TNF-cell receptor complex [18]. Infliximab has a variable mouse (murine) region with a constant human region in contrast to adalimumab, a completely humanized antibody. Etanercept is a soluble TNF receptor that inactivates both TNF-α and β by binding to its soluble forms [18]. Figure 2 below illustrates this.

Infliximab and adalimumab are associated with the formation of neutralizing antibodies after prolonged usage; etanercept is not associated with the formation of neutralizing antibodies with prolonged use [18].

Use of Biologic Agents in Extra-pulmonary Sarcoidosis

Several recent observational studies have described the use and efficacy of anti-TNF agents in extra-pulmonary sarcoidosis in the organ systems (Table 1).

**Table 1:** Observational Studies Describing Use of Anti-TNF Agents in Extra-pulmonary Sarcoidosis

Author	Year of publication	Study type/no. of patients	Purpose of the study	Drugs used/organ-system	Results/conclusion
Rosenthal et al. [32]	2019	Retrospective chart review; n=28	To determine the efficacy of immunosuppressant therapy in cardiac sarcoidosis.	Methotrexate + adalimumab in cardiac sarcoidosis	Combination therapy of Methotrexate and Adalimumab yielded noteworthy suppression of myocardial inflammation.
Gelfand et al.[28]	2017	Retrospective chart review; n=66	To describe clinical & imaging responses of patients with refractory neurosarcoidosis to infliximab.	Infliximab in refractory neurosarcoidosis	Infliximab yielded notable improvement in clinical and imaging evaluation in neurosarcoidosis.
Zhou et al. [3]	2017	Retrospective chart review; n=64	To describe the clinical characteristics & treatment of patients with bone sarcoidosis.	Infliximab in refractory bone sarcoidosis	Infliximab was effective in aggressive bone sarcoidosis refractory to steroids &Methotrexate
Heidelberger et al. [1]	2017	Retrospective observational; n=46	To determine the efficacy and safety of biologic agents in cutaneous sarcoidosis.	Infliximab in cutaneous sarcoidosis	Biologic agents yielded 84% positive response rate with the resolution of cutaneous nodules
Banse et al.[37]	2013	Retrospective study; n=10	To determine the efficacy of anti-TNF agents in sarcoidosis of joints.	Infliximab, adalimumab, etanercept in articular sarcoidosis	Biologic agents are not effective in articular sarcoidosis.
Hostettler et al.[9]	2012	Retrospective chart review; n=28	To determine the efficacy of infliximab in extra-pulmonary sarcoidosis.	Infliximab in cutaneous, cardiac and neurosarcoidosis	Long term infliximab therapy more beneficial in multi-organ sarcoidosis involvement.
Erckens et al.[31]	2011	Prospective cohort study; n=26	To determine the efficacy of Adalimumab in uveitis due to sarcoidosis.	Adalimumab In sarcoid uveitis	Adalimumab yielded notable suppression of inflammatory signs in 85% of respondents.
Aguiar et al.[36]	2011	Prospective case series; n=10	To determine the efficacy of infliximab in extrapulmonary sarcoidosis	Infliximab in extra-pulmonary sarcoidosis	Infliximab was particularly effective in cutaneous and neurologic sarcoidosis.

**Figure 3:**
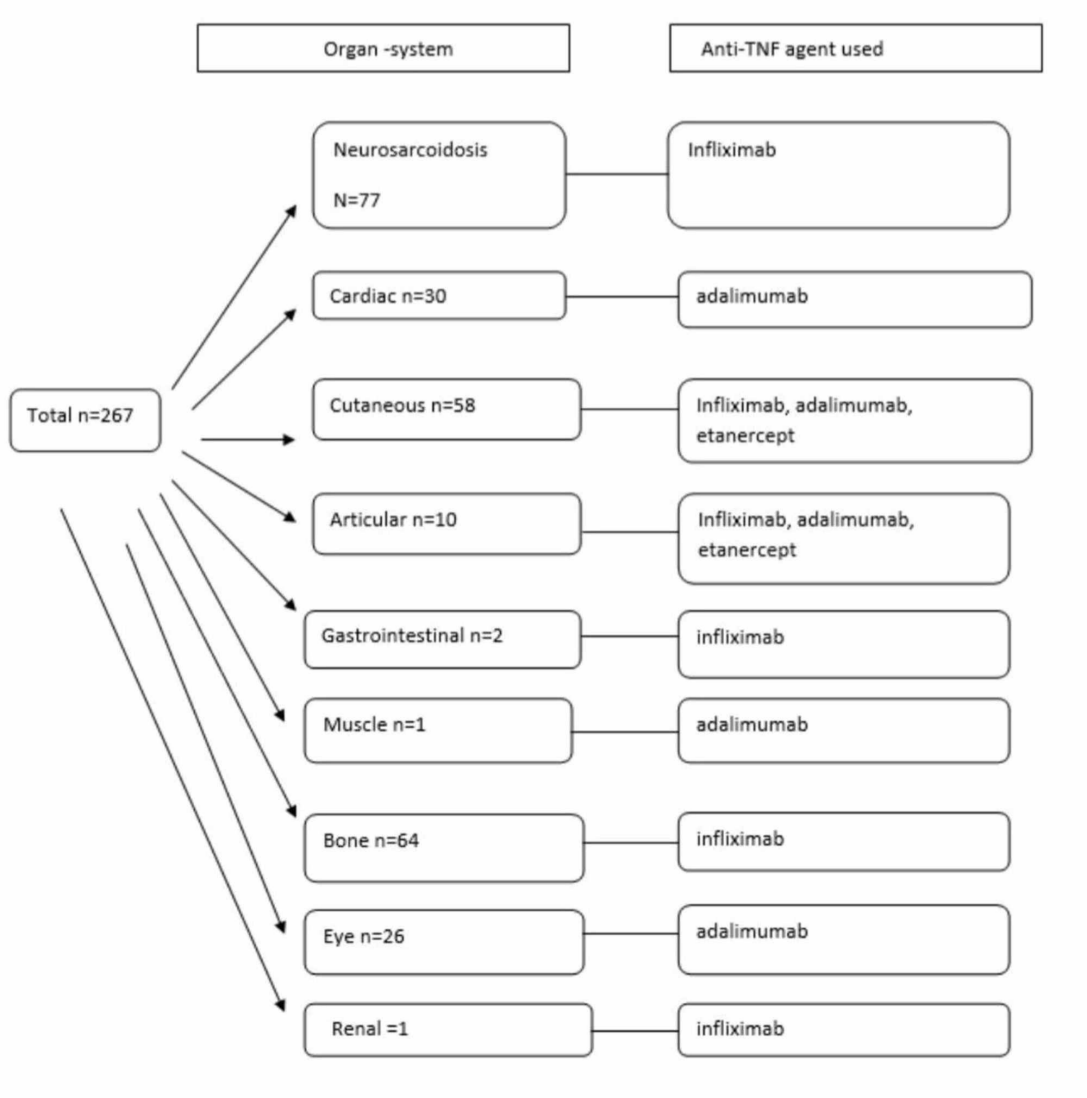
Organ-systems of Involvement

We summarize the organ-systems affected in the patients with extra-pulmonary sarcoidosis (including case series/reports) alongside with the anti-TNF agent used for treatment in Figure 3.

Our selected studies describe the use of infliximab in neural, cutaneous, gastrointestinal, articular, renal, and bone sarcoidosis. Adalimumab was used predominantly in cardiac, cutaneous, articular, muscle, and ocular sarcoidosis. Etanercept was used only in cutaneous and articular sarcoidosis.

Methods by which studies assessed for the efficacy of individual anti-TNF agents include the extent of regression of lesions on imaging, steroid-sparing property, or improvements on clinical evaluation. The studies, however, fall short of making direct head to head comparisons in efficacy between the three agents.

Infliximab

The reported cases, case series, and observational studies on extra-pulmonary sarcoidosis wherein infliximab were used suggest that infliximab is effective. Gelfand et al. performed a retrospective chart review on 66 patients demonstrating that patients with neurosarcoidosis refractory to steroids on infliximab therapy had significant improvement in clinical and imaging evaluations [28]. Two case reports on neurosarcoidosis had similar positive outcomes with infliximab [16,27]. Zhou et al., in an observational study with the largest sample size on patients with bone sarcoidosis showed infliximab to be highly effective based largely on PET/CT results on follow up [3]. Heidelberger et al. showed an 84% positive response rate amongst participants with cutaneous sarcoidosis on monotherapy with infliximab [1]. Studies by Aguiar et al. and Hostettler et al. included patients with multi-system involvement, and infliximab therapy was more effective in achieving disease remission in patients with multi-organ involvement compared to patients with only pulmonary sarcoidosis [9,36]. Cases of sarcoidosis in the gastrointestinal tract and a renal transplant were managed successfully with infliximab [15,27]. Infliximab was ineffective in articular sarcoidosis [37].

Adalimumab

There is literature describing the use of adalimumab in extra-pulmonary sarcoidosis refractory to steroids. These include studies by Rosenthal et al., a retrospective study involving 28 patients with cardiac sarcoidosis wherein adalimumab was successfully used in combination with methotrexate to achieve notable suppression in myocardial inflammation [32]. The study recommends a strategy of long term combination therapy of methotrexate and adalimumab to achieve disease quiescence in cardiac sarcoidosis. Patients with cardiac sarcoidosis may also present with arrhythmias due to the involvement of the conducting system of the heart. In a case report by Theodore et al., adalimumab therapy was used to achieve a reversion to sinus rhythm at the time of administering the third dose [21]. Erkens et al. followed up 26 sarcoid uveitis patients on adalimumab [31]. Results at the 12-month endpoint showed an 85% response rate with a reduction in both intra-ocular inflammation and other clinical indicators of disease activity such as fatigue. One study reports that adalimumab was successfully used in treating sarcoid myositis [19]. As with infliximab, adalimumab has not been used in treating articular sarcoidosis successfully [37].

Etanercept

There is insufficient evidence supporting the use of etanercept as an effective steroid-sparing agent in sarcoidosis. Marques et al. describe a case of peripheral neuropathy successfully treated with etanercept [18]. The patient had discontinued infliximab and adalimumab in succession after developing neutralizing antibodies to these agents but responded to etanercept. The study suggests that etanercept may be considered when there is treatment failure with other biologic agents.

In summary, medium level literature evidence pooled from observational studies, and case series suggest that infliximab, adalimumab, and etanercept are widely used either as monotherapy or in combination with methotrexate in extra-pulmonary sarcoidosis refractory to steroids with infliximab having the broadest range of organ system use. Although there are no head-to-head comparisons of their efficacy, the studies show that infliximab and adalimumab have been used extensively with positive outcomes in various multi-organ systems except in articular sarcoidosis. Infliximab has even been noted to give better results in extra-pulmonary locations compared to patients with predominantly pulmonary sarcoidosis [9,36]. Etanercept has the least supporting evidence for usage in extra-pulmonary sarcoidosis, and outcomes have been discouraging.

Use of Biologic Agents in Adult Non-infective Uveitis

Non-infective uveitis (NIU) is a sight-threatening condition frequently associated with autoimmune disorders. Although the inciting antigen is often not known, TNF plays a key role in the mediation of the inflammatory response. Causative autoimmune disorders include Behcet's disease, ankylosing spondyloarthropathy, sarcoidosis, and juvenile idiopathic arthritis.

There is high-quality evidence supporting the use of adalimumab in treating adult NIU such as Leal et al. [11], a systematic review with meta-analysis of three randomized clinical controlled trials (VISUAL I and II, and Foster et al.), with overall 458 enrolled patients: n=230 in the anti-TNF arm and n=228 in the control arm [38-40]. The purpose of the study was to emphasize the efficacy of adalimumab in adult NIU. Therapeutic endpoints used to determine efficacy included the ability to mitigate intra-ocular inflammation and prevent vision loss as determined by improved best-corrected visual acuity (BCVA), as well ability to decrease the occurrence of flares of uveitis. The first two studies (VISUAL I and II) were multinational randomized, placebo-controlled trials focused on the safety profile and efficacy of adalimumab. They showed that patients in the adalimumab arm had significant improvements in BCVA compared to the control arm [38,39]. Eligible patients in these studies were enrolled in VISUAL III, a phase III, open-label, clinical trial extension. The results showed that active uveitis patients who continued on adalimumab were less likely to have disease reoccurrence and achieved disease quiescence [41]. The third RCT included in the systematic review, Foster et al., with a sample size of 20, assessed the efficacy of etanercept in adult NIU [40]. Still, they showed that etanercept did not result in the preservation of visual acuity any more than the placebo group [40]. The US Food and Drug Administration (FDA) eventually approved adalimumab as the only anti-TNF agent for use in adult NIU based on the findings of the VISUAL I, II, and subsequently, VISUAL III studies. Hiyama et al., in 2019, published a report of two cases of Behcet's uveitis treated successfully with adalimumab after it became refractory to steroids [13].

What Is the Comparative Efficacy of Infliximab and Adalimumab in Refractory Adult Non-infectious Uveitis?

A study to compare infliximab and adalimumab in terms of efficacy and safety profile in uveitis was published in 2016 and conducted by the French uveitis network [30]. It was a retrospective observational study with a sample size of 60 patients with NIU refractory to steroids. The median age of participants was 31 years. The purpose of the study was primarily to compare the efficacy of infliximab and adalimumab at a six-month endpoint. A complete response is defined by the degree of reduction in both intra-ocular inflammation and initial steroid dosage at the start of treatment. The secondary goal of the study was to determine the factors that favor a complete response. About 61% (n=98) and 39% (n=62) of study participants were treated with infliximab and adalimumab, respectively. Results show that infliximab and adalimumab are equivalent in terms of efficacy. The secondary outcome showed that in a subset of patients with NIU due to Behcet's, the number of flares before the commencement of biologic correlates with a higher likelihood of complete resolution.

In summary, even though only adalimumab is approved for use in treating adult NIU, infliximab still has extensive off-label usage, and a study has shown that both are equally efficacious [30]. A randomized clinical trial has demonstrated that etanercept has no benefit in uveitis [40].

Role of Biologic Agents in Pediatric NIU

Pediatric uveitis is a potential cause of blindness in children. It is commonly caused by juvenile idiopathic arthritis (JIA) and typically occurs anteriorly (chronic anterior uveitis- CAU). Steroids are the standard first-line treatment for this condition [10]. The cornerstone for long term treatment, however, is methotrexate, as prolonged steroid therapy is undesirable, and methotrexate has been reported to have response rates of up to 73% [29].

**Figure 4:**
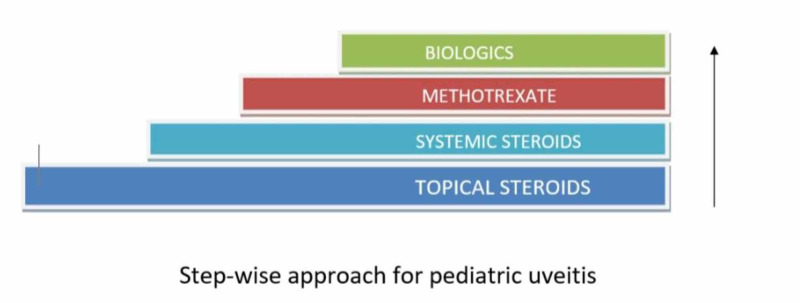
Step-wise approach for Pediatric Uveitis Treatment

There are no recently published randomized clinical trials to guide the timing of biologic agents, but there is data from recent observational studies and case reports to support its use [14,20,24,29,30,33-35]. Figure 4 below describes a step-wise approach in treating pediatric uveitis.

A study published in 2019 performed on a cohort of children with CAU concludes by supporting the use of biologic agents in severe uveitis or where patients do not tolerate methotrexate [29]. In this study, children with severe uveitis who commenced biologic agents earlier had better visual outcomes. The study also suggests that children with idiopathic CAU require biologics earlier compared to CAU caused by juvenile idiopathic arthritis.

There were two observational studies comparing infliximab and adalimumab in terms of efficacy in pediatric CAU [30,33]. Both argue that although infliximab and adalimumab are effective in achieving remission, adalimumab is better at maintaining remission in CAU. Zannin et al. conducted a prospective cohort study on follow up data at one year from an Italian registry and found that children on adalimumab had higher remission rates and likelihood to remain on remission at one year follow up [33]. The second study had similar outcomes but measured remission rates at three years, and adalimumab was more effective than infliximab at maintaining remission at three years [30]. The first case report of adalimumab use in a child less than three years was published in 2014 by La Torre et al. [14]. The patient, a two-year-old girl with refractory JIA-uveitis, had developed growth retardation from chronic steroid use and responded to adalimumab therapy. Perhaps the most substantial study to demonstrate the efficacy of biologic agents in pediatric uveitis is a systematic review conducted by Simonini et al. [12], which pooled data from several observational studies on 229 children. The results showed no difference between infliximab and adalimumab in the proportion of respondents with positive treatment outcomes. They concluded that both agents have similar efficacy but are superior to etanercept. However, upon achieving remission, a higher proportion of children on adalimumab maintained long term remission.

To summarize, several recent observational studies and a systematic review support the use of biologic agents in severe pediatric uveitis to preserve vision or when methotrexate is not used due to side effects. Adalimumab is more effective than infliximab at achieving uveitis remission in the long term. Etanercept is not an effective agent in chronic pediatric uveitis.

The Safety Profile of Biologic Agents

Biologic agents are well tolerated as monotherapy or add-on therapy in sarcoidosis or uveitis. The most common adverse events seem to be localized administration site reactions, infections (respiratory and gastrointestinal), and hypersensitivity reactions [1,5,9,20,30,33,37,41]. Patients concomitantly on biologics and steroids have a higher risk of infections [1].

There is, however, a black box warning for reactivation of latent tuberculosis and invasive mycotic infections [41]. These are feared complications of treatment that have occurred during drug surveillance. Screening for latent tuberculosis should be done before commencing therapy. The safety profile for adalimumab and infliximab in uveitis are similar and can be tolerated in the long term [33]. Adalimumab can be tolerated in children less than three years [14].

Limitations

There were no randomized clinical trials within the period of study that compared the efficacy of anti-TNF agents to placebo or each other in refractory extra-pulmonary sarcoidosis.

## Conclusions

Extra-pulmonary sarcoidosis and chronic uveitis are inflammatory conditions that both have tumor necrosis factor as a common denominator in their pathogenesis. Our review was focused primarily on determining the extent of the use of biologic agents in these conditions. The secondary aim of the review was to compare the efficacy and safety profile of the common biologic agents used for their management. Our findings in both conditions show that infliximab and adalimumab are used as alternatives to steroids. In sarcoidosis, compared to adalimumab, infliximab has been used successfully in more organ-systems. Infliximab has been used mostly for neural, gastrointestinal, renal, and bone sarcoidosis, while adalimumab has principally been used for treatment in cardiac, muscle, and ocular sarcoidosis. Both agents have been used for cutaneous and albeit unsuccessfully, in articular sarcoidosis. In terms of efficacy of use, both agents are similar and are superior to etanercept, which has limited use. In chronic pediatric uveitis, adalimumab is superior at maintaining long term disease remission. In terms of safety profile, biologic agents are mostly well tolerated but require surveillance due to the potential for life-threatening systemic infections and reactivation of latent tuberculosis that may occur. Due to the rarity of extra-pulmonary sarcoidosis in the population, there were no randomized clinical trials within the period of study to make direct comparisons between agents. More randomized clinical trials are required to inform better evidence-based use of these agents by clinicians in the future.
